# Effect of Modifier Form on Mechanical Properties of Hypoeutectic Silumin

**DOI:** 10.3390/ma16155250

**Published:** 2023-07-26

**Authors:** Tomasz Lipiński

**Affiliations:** University of Warmia and Mazury in Olsztyn, 10-719 Olsztyn, Poland; tomaszlipinski.tl@gmail.com or tomekl@uwm.edu.pl

**Keywords:** Al alloys, hypoeutectic silumin, modification, modifier

## Abstract

Aluminum–silicon alloys require modification due to their coarse-grained microstructures and resulting low strength properties. So far, research into the modification process has focused on the use of various chemical components and technological processes, the tasks of which are to refine the microstructure and, thus, increase the mechanical properties of the alloy. In this paper, the answer to the question of whether the form of the modifier influences the modification effect of the hypoeutectic silumin will be found. The tests were carried out using the popular silumin AlSi7Mg. To answer our research question, the alloy was modified under comparable conditions using the following elements: Ti, B, and master alloys AlTi1.5 and AlB1.5. Modifiers in the form of Sr and master alloy AlSr1.5 were also used. All mentioned modifiers were produced and introduced into the liquid alloy in the form of a powder and a rod. Master alloys AlSr1.5 were also produced via cooling from the liquid state through cooling in air and the second variant at a speed of 200 °C/s (in the form of powder and a thin strip). The microstructure and mechanical properties were analyzed based on the following measures: tensile strength, elongation, and hardness of silumin. Based on the conducted research, it was found that the form of the modifier also affects the modification effect visible in the form of changes in the microstructure and mechanical properties. For the powder-modified alloy, greater fineness in the eutectic phase (α and B phases) and an increase in all analyzed mechanical properties were obtained.

## 1. Introduction

Silumins are one of the most commonly used metal casting alloys. Regardless of the silicon content in the raw state, they have coarse grains formed of the β phase, which is a solid solution of aluminum in silicon, and the α phase, which is a solution of silicon in aluminum. Silumins that contain up to 12.6% Si are referred to as hypoeutectic. The primary phase formed using the liquid is the α phase, which enriches the liquid with silicon, and, at a temperature of 577 °C, the rest of the liquid phase crystallizes into eutectic form (α and β phases). The eutectic β phase occurs in the form of grains and needles. These divisions are relatively large. Since the β phase is hard and not very plastic in nature, when it is present in the eutectic, it creates features similar to microstructural notches that reduce the mechanical properties of the alloy [1,2,3,4,5,6,7,8,9,10]. In this form, the alloy has low strength and ductility, and its hardness depends on the eutectic morphology of the β phase. Considering the initial use of silumins, research has been carried out to increase their functional properties. Currently, several ways of increasing their mechanical properties are known. Increasing their mechanical properties can be achieved using various technological factors [11,12,13,14,15,16,17,18]. The mechanical properties of the alloy can be shaped by the use of different cooling rates, pressure casting, thixotropic casting, mechanical vibrations, ultrasonic waves, etc. [19,20,21,22,23,24,25,26]. All of these methods of production have found application in industry. An equally popular way to increase the mechanical properties of alloys is modification with elements, chemical compounds, and chemical mixtures. In the past, many methods of introducing modifiers into liquid alloys were developed. Modification can be carried out in a crucible or casting ladle; alternatively, it can be directly carried out in the casting mold. Methods of carrying out modifications within the casting mold that have gained popularity include the imconod and inmold methods [27,28], which are also called flow modification methods. The modification of silumins involves introducing special additives (modifiers/changers) into the metal bath, which significantly affect degree of supercooling, changing the course of the crystallization phenomena and allowing them to be controlled. Changes in the mechanical properties of the solidified metal after modification do not result from a change in the chemical composition of the alloy, instead resulting from a change in the conditions of its crystallization. Although the modification of a liquid alloy is a commonly used procedure today, the essence of the process and the definition of the procedure itself have not been precisely and unambiguously defined. There are many hypotheses that aim to explain this phenomenon [29,30,31,32].

Commonly used industrial modifiers are most often based on the modifying action of strontium or sodium. Sr-modified alloys have better castability than Na-modified alloys, most likely due to their thick layer of oxides (the so-called elephant skin). Both modifiers cause porosity in alloys when casting into sand molds. There are a number of papers that describe the effect of modification after adding various modifiers, including Sr [33,34,35,36], Na [2,37,38]; Ti and B [39,40,41,42,43,44,45,46]; Li [47]; Ba, Ca, and Y [48,49]; Sc [50]; Y [51]; Sb [52]; and others. For grain refinement in Al casting alloys, alloys based on titanium and boron can be used. An example of this approach is the AlTi5B1 alloy.

The topic of the modification of hypoeutectic silumins is widely described in the literature. From time to time, however, research results are published that show new solutions, thus confirming the desirability of performing further work in this area. In recent years, an ecological approach that modifies silumins using the homogeneous method has been shown. This method showed the possibility of controlling the microstructure and mechanical properties of silumin using a modifier with a chemical composition similar or identical to that of the treated alloy [53].

By analyzing the literature reports and comparing them to our own observations, we considered whether it is possible to modify, and, thus, change, the microstructure and mechanical properties of hypoeutectic silumin by controlling the form of the modifier. This research focus is justified as we confirmed that the order in which the chemical components (elements that constitute the modifying mixture) are introduced into the alloys is important for the modification effect of the Al–Si alloys.

## 2. Materials and Methods

The tests were carried out on hypoeutectic silumin AlSi7Mg, and the chemical composition is shown in Table 1.

**Figure 1:**
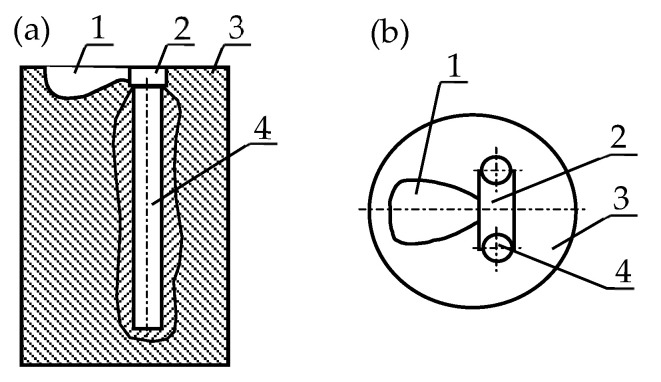
The diagram of the casting mold: 1—pouring cup, 2—sprue, 3—mold, 4—casting (samples); (**a**) section, (**b**) top view.

Silumin and all master alloys were melted using an electric furnace. Al_2_O_3_ ceramic crucibles were used. For the modification process, 750 g of silumin was used per one melt. The alloy that remained after the test was removed from the crucible, and the crucible was cleaned before the next melting test. In each series of tests, the alloy was modified at 780 °C and 20 min. From the modification temperature, the alloy was poured into a casting mold. The diagram of the casting mold is shown in Figure 1.

Before modification of the silumin, a test melt was carried out by casting a pure alloy into a mold, for which process metallographic tests and mechanical properties tests were carried out.

In the first series of tests, titanium and boron were used to modify the AlSi7Mg alloy. Titanium was used in the form of a powder with a purity of >98.5% Ti (Ti pow.), and boron was used in the form of a powder with a purity of >95% (B pow.), in the form of boron puriss p.a. crystalline pieces (B piec.), and in the form of pellets produced via pressing into a special form using a hydraulic press and boron in the form of powder. A pellet with a diameter of 3 mm and a length of about 10 mm was, thus, obtained (B pell.).

In the second series, specially prepared master alloys were introduced into the liquid AlSi7Mg alloy. Master alloys were prepared by introducing 1.5% Ti or 1.5% B into the liquid silumin. The master alloys obtained—AlTi1.5 and AlB1.5—were cast into crucibles in the form of a rod with a diameter of 3 mm (using the suction method). The cast rods were used to directly modify the silumin (AlTi1.5 rod and AlB1.5 rod), while the powder was obtained via mechanical grinding of the rods. The modifications in both the first and second series were carried out by calculating the amounts of Ti and B at the level of 0.01% based on the weight of the treated silumin. The literature reports the use of titanium and boron to modify hypoeutectic silumin in various amounts [54,55,56]. Ready-made technical alloys are usually used, e.g., AlTi5B1 (5% Ti, 1% B) [57,58]. In this study, we decided to use the average concentration of titanium and boron in the master alloy (the concentrations were the same for both components).

In the third series, strontium modification and strontium-based master alloys were carried out. Master alloys in the form of rod (AlSr rod) and powder (AlSr pow.) were produced in a similar manner to the master alloys produced in the second series. Master alloys cooled at 200 °C/s were produced in a similar manner; however, they poured not into a crucible, but onto a rotating copper pad, before being mechanically ground ((AlSr)c) or 3-millimeter-wide strips ((AlSr rod)c), which were introduced into the liquid alloy. The amount of strontium and master alloy was converted into the mass content of the modified alloy, meaning that the amount of Sr was at a constant level of 0.03%. The cooling rate of the modifier was adopted on the basis of [53].

The static tensile test was carried out using the ZD10 machine on five-fold cylindrical samples that each had a measuring diameter of 6 mm, according to EN ISO 6892-1:2016 [59]. Two samples were tested for each heat level. Brinell hardness was tested on the heads of samples prepared for a static tensile test, in accordance with ISO 6506-1:2014 [60], using a ball with a diameter of 2.5 mm, a load of 306.5 N, and a load time of 20 s in the HPO 250 hardness tester. Three measurements were taken on each sample. The arithmetic mean of the measurements was used for the analysis. Metallographic examinations were carried out using the Olympus IX70 optical microscope via DLTCamViwer after the samples had been etched with the Mi8Al reagent. Phase analysis was performed using an X-ray Phaser Bruker diffractometer through Difrac EVA and HighScore Plus software and a crystallographic database.

## 3. Results and Discussion

The mechanical properties of the tested silumin cast into the mold Figure 1 were as follows: Rm = 137 MPa, A = 0.7%, and H = 51 HB.

**Figure 2:**
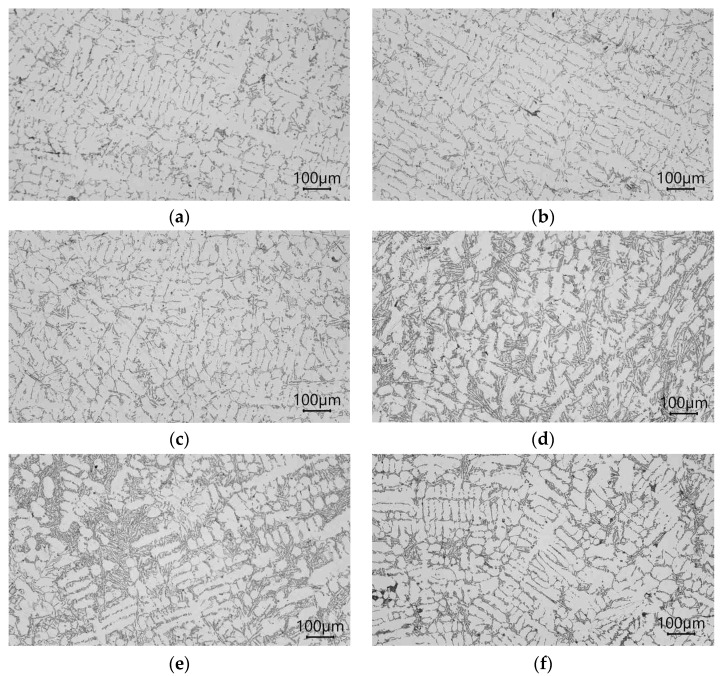
Microstructure the AlSi7Mg alloy (**a**) without and after modification with the following components: (**b**) Ti as a powder; (**c**) Ti as a rod; (**d**) B as a powder; (**e**) B as pieces; (**f**) B as pellets.

The microstructure of the AlSi7Mg alloy without and after modification via the addition of Ti and B to the liquid alloy in the form of powder, pieces, and pellets is shown in Figure 2.

**Figure 3:**
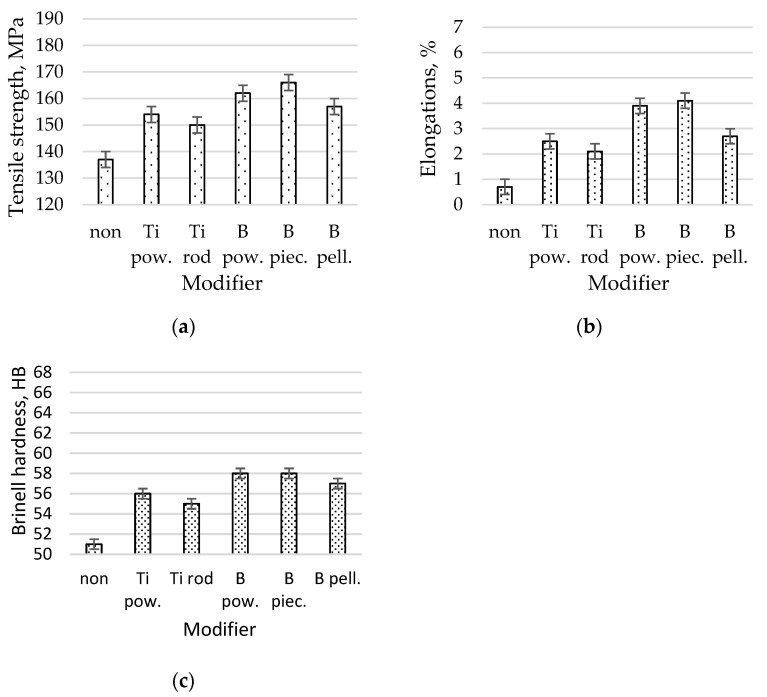
Mechanical properties of the AlSi7Mg alloy without and after modification with Ti as a powder (Ti pow.), Ti as a rod (Ti rod), B as a powder (B pow.), B as a pieces (B piec.), and B as a pellet (B rod); (**a**) tensile strength, (**b**) elongations, (**c**) Brinell hardness.

The mechanical properties the AlSi7Mg alloy without and after modification with Ti as a powder, Ti as a rod, B as a powder, B as a pieces, or B as a pellet are shown in Figure 3.

The microstructure of the unmodified alloy is composed of eutectic silumin (α and β phases) against the background of the α phase, as shown in Figure 2a. Eutectic silicon has a plate shape, which is visible on the metallographic microsection in needle form. The distribution of these needles is random throughout the silumin volume. An alloy with such a microstructure has low mechanical properties (tensile strength Rm = 137 MPa, elongation A = 0.7%, and hardness 51 HB), as shown in Figure 3. After introducing titanium powder into the alloy, increases in tensile strength of 12% to 154 MPa, elongation of 36% to 2.5%, and hardness of 4 HB were noted, and the phase morphology was slightly changed β (Figure 1b). Modification with titanium in the form of a rod caused similar changes in the microstructure (Figure 2c), though slightly lower mechanical properties were obtained Rm = 150 MPa, A = 2.1%, and H = 55) than via powder modification (Figure 3). After introducing powdered boron into the alloy, greater fineness of the eutectic β phase was observed for boron than for titanium (Figure 2d). It is already possible to identify the eutectic β phase that occurs in the precipitates in the interdendritic spaces, which was formed as the first α phase. An alloy with tensile strength of Rm = 162 MPa, elongation of A = 3.9%, and hardness of 58 HB was obtained. Even greater fragmentation of the β phase was obtained after the addition of boron in the form of pieces (Rm = 166 MPa, A = 4.1%, H = 58 HB). It can be seen that an increase in the degree of modification results in increase in Rm of 21% to 166 MPa, A of 486% to 4.1%, and hardness of 6 HB (relative to the alloy without modification), and the β phase occurs in the form of finer precipitates, which tend to increase the efficiency modification to equiaxial dimensions (Figure 2e) [2,3,4,5]. The addition of boron in the form of pellets into the alloy resulted in an increase in mechanical properties in relation to the starting alloy (Rm = 150 MPa, A = 2.7%, and H = 57 HB), though the strength parameters were not as high as they were after the addition of boron in the form of pieces (Figure 3); this discrepancy was reflected in the microstructure (Figure 2f). Fragmentation of the eutectic silumin (α and β phases) was obtained, though there was a clear increase in the size of the dendrites of the primary α phase.

**Figure 4:**
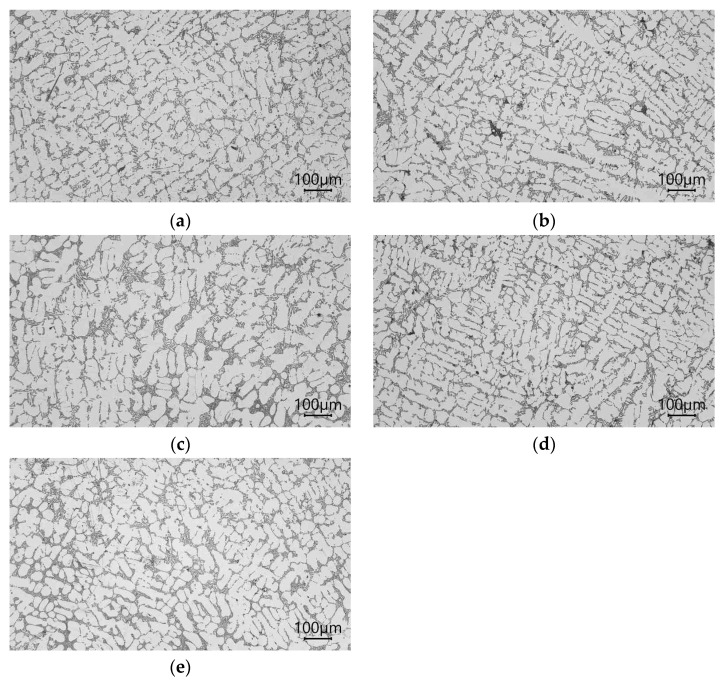
Microstructure the AlSi7Mg alloy with the following properties: (**a**) AlTi1.5 as a powder, (**b**) AlTi1.5 as a rod, (**c**) AlB as a powder, (**d**) B as a pellet, (**e**) AlTi1.5B1.5 as a rod.

The microstructures of the AlSi7Mg alloy with AlTi1.5 as a powder, AlTi1.5 as a rod, AlB as a powder, B as a pellet, and AlTi1.5B1.5 as a rod added to the liquid alloy are shown in Figure 4.

**Figure 5:**
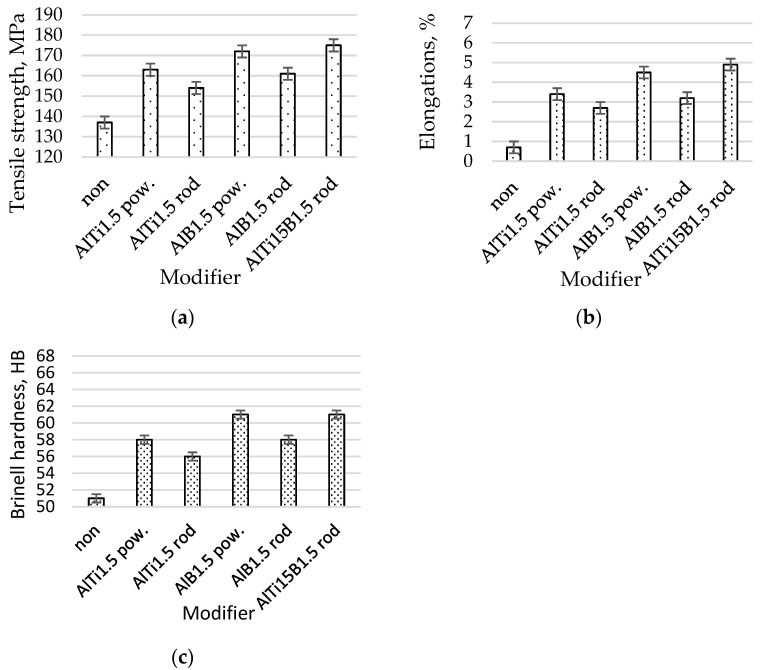
Mechanical properties the AlSi7Mg alloy with AlTi1.5 as a powder, AlTi1.5 as a rod, AlB1.5 as a powder, AlB1.5 as a rod, and AlTi1.5B1.5 as a rod; (**a**) tensile strength, (**b**) elongations, (**c**) Brinell hardness.

**Figure 6:**
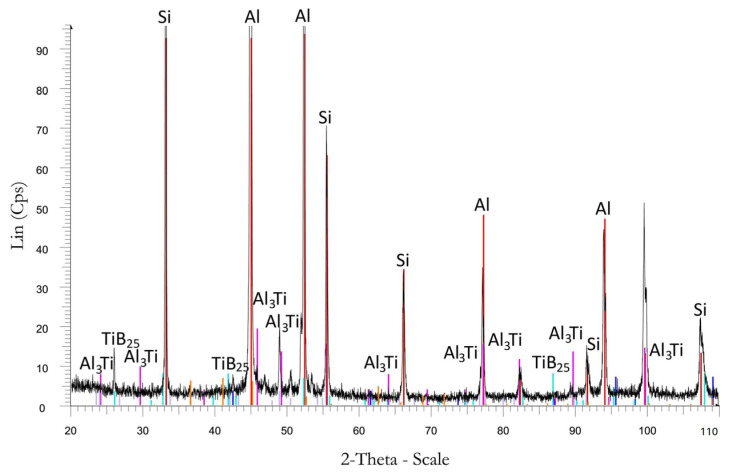
XRD of AlSi7Mg alloy with AlTi1.5B1.5.

After modification of the master alloy based on Ti and B into analogous modifier forms, a greater fragmentation of the β eutectic phase than those of Ti and B added separately in the form of alloying elements was obtained (Figure 4). All microstructures for this test series were partially modified. For AlSi7Mg with AlTi1.5 in powder form, properties of Rm = 163 MPa, A = 3.4%, and 58 HB were achieved (Figure 5). After the addition of the AlTi1.5 master alloy in the form of a rod, a strength clearly lower than that of the same powder modifier was noted. Properties of Rm = 163 MPa, A = 2.7%, and 56 HB were recorded (Figure 5). These results correlate with the degree of fragmentation in the β phase (Figure 4b). After the addition of AlB1.5 in the form of powder, clear increases in the fineness of the eutectics (α and β phases) (Figure 4c) and mechanical properties resulting from the fragmentation microstructure were noted. Strength increased by 26% to Rm = 172 MPa; elongation increased by more than 900% to A = 4.5%, relative to that of the not modified alloy; and hardness increased by up to 61 HB. After introducing AlB1.5 into the silumin in the form of a rod, in relation to the starting alloy, increases in Rm of 18% to 161 MPa, elongation of 46% to A = 3.2%, and hardness of 7 HB were noted. These values are lower than those of the modifier of the same chemical composition added in the form of powder. Thicker precipitations of eutectic (α and β phases) and dendritic α phases were also noted (Figure 4d). For the modifier composed of both titanium and boron AlTi1.5B1.5 in the form of a rod, even greater fragmentation of eutectic silumin (α and β phases) (Figure 3e) and higher mechanical properties were obtained [43,54,55,56,57,58]. These results prove the possibility of further increasing the mechanical parameters of the alloy. An increase in mechanical properties was noted in relation to the starting alloy: tensile strength increased by 28% to Rm = 175 MPa, elongation increased by 600% to A = 4.9%, and hardness increased by 10 HB to 61 HB (Figure 5). The XRD record for AlSi7Mg with AlTi1.5B1.5 in the form of a rod, as shown in Figure 6, confirms the presence of phases in the alloy with the participation of both titanium and boron, which confirms the transfer of the introduced additives into the alloy.

The mechanical properties of the AlSi7Mg alloy without and after modification with master alloys AlTi1.5 as a powder, AlTi1.5 as a rod, AlB1.5 as a powder, AlB1.5 as a powder, and AlB1.5 as a rod are shown in Figure 5.

The X-ray diffraction of AlSi7Mg with AlTi1.5B1.5 is shown in Figure 6.

**Figure 7:**
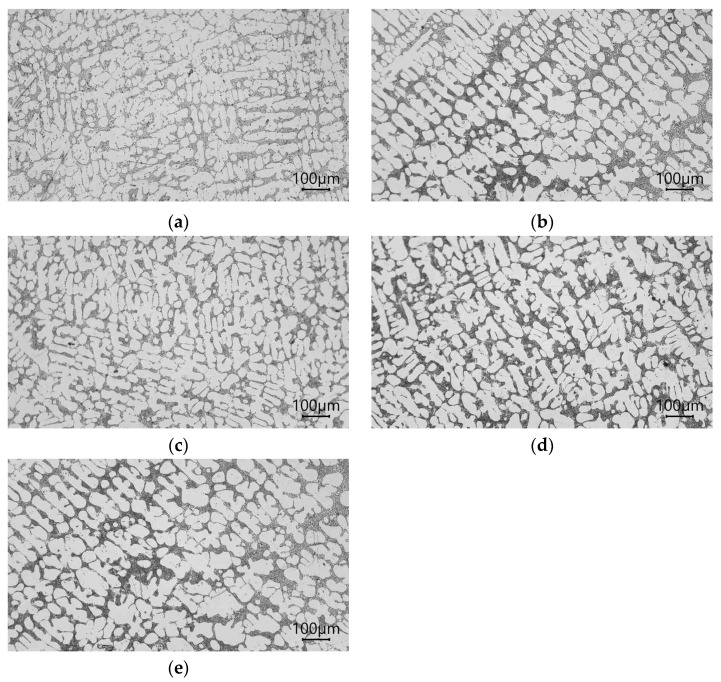
Microstructures of the AlSi7Mg alloy with the following properties: (**a**) 0.03% Sr as a powder; (**b**) master alloy AlSr1.5 as a powder; (**c**) master alloy AlSr1.5 as a rod; (**d**) master alloy AlSr1.5 cooled at 200 °C/s as a powder; (**e**) master alloy AlSr1.5 cooled at 200 °C/s as a rod.

The microstructures of the AlSi7Mg alloy with Sr as a powder, the master alloy AlSr1.5 as a powder, the master alloy AlSr1.5 as a rod, the master alloy AlSr1.5 cooled at 200 °C/s as a powder, and the master alloy AlSr1.5 cooled at 200 °C/s as a rod are shown in Figure 7.

**Figure 8:**
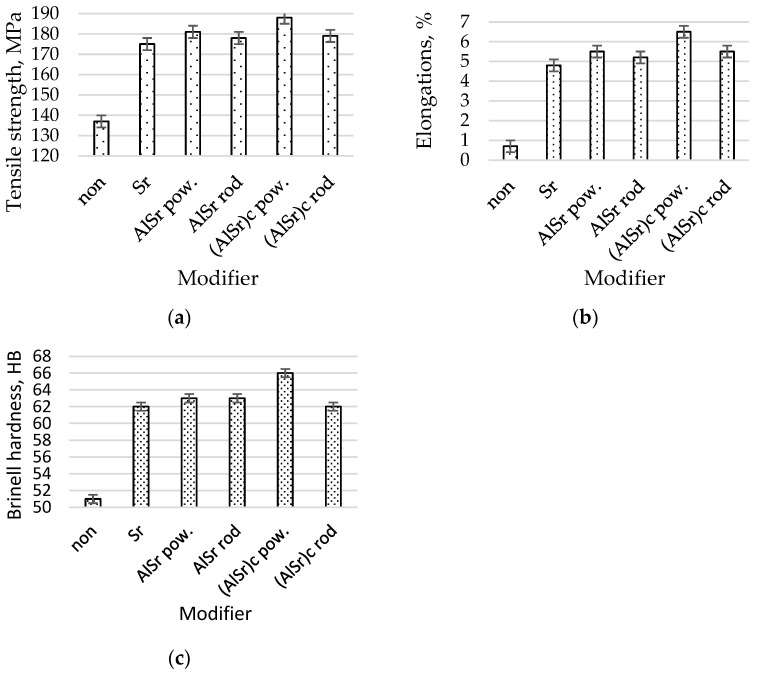
Mechanical properties of the AlSi7Mg alloy with 0.03% Sr as a powder (Sr), master alloy AlSr1.5 as a powder (AlSr pow.), master alloy AlSr1.5 as a rod (AlSr rod), master alloy AlSr1.5 cooled at 200 °C/s as a powder ((AlSr)c), and master alloy AlSr1.5 cooled at 200 °C/s as a rod; (**a**) tensile strength, (**b**) elongations, (**c**) Brinell hardness.

The mechanical properties of the AlSi7Mg alloy without modification and with Sr as a powder, the master alloy AlSr1.5 as a powder, the master alloy AlSr1.5 as a rod, the master alloy AlSr1.5 cooled at 200 °C/s as a powder, and the master alloy AlSr1.5 cooled at 200 °C/s as a rod are shown in Figure 8.

The microstructure of AlSi7Mg with Sr is shown in Figure 7a. The eutectic silumin (α and β phases) is fine. The primary α phase also has thin dendrite arms. This microstructure is modified. This microstructure is reflected in the following mechanical properties (Figure 8): Rm = 175 MPa, A = 4.8%, and H = 62 HB; thus, there was an increase in these parameters in relation to the starting alloy Rm of 28%, A of 585%, and H of 22%. After adding the AlSr master alloy in the form of powder into the liquid alloy, further increases in the mechanical properties of 6 MPa and 0.7% were observed in relation to the strontium-modified alloy (Figure 8), which were reflected in the finer eutectic and directional arrangements of the axes of the main α-phase dendrites (Figure 7b). After adding AlSr in the form of a rod, slight decreases in mechanical properties (Rm = 178 MPa, A = 5.2%, and H = 63 HB) were noted in relation to the modifier in the form of powder (in principle, it was on the verge of being an error, although that it was confirmed in two parameters—Rm decreased by 3 MPa and A decreased by 0.3%—provides grounds for considering such a possibility). The decrease in these parameters was small. This result may be due to the good modifying efficiency of strontium, which partly offsets the lower efficiency of modification using a rod modifier. After silumin modification using a modifier produced via rapid cooling (according to assumptions [53]), further fragmentation of the eutectic (α and β phases) phase was noted (Figure 7d,e); however, the class of the modifier in the form of a rod was noted to have larger sizes in the primary α phase. An increase in strength properties was also noted (Figure 7). After being added into the silumin master alloy, AlSr1.5% cooled at a speed of 200 °C/s in the form of a powder, and it had the following mechanical properties: Rm = 188 MPa, A = 6.5%, and H = 66 HB. After using the same modifier in the form of a rod, we obtained the following mechanical properties: Rm = 179 MPa, A = 5.5%, and H = 62 HB (Figure 8). Thus, after modifying the Al7SiMg alloy using the modifier AlSr1.5 in the form of powder, a higher efficiency of modification was obtained, resulting in greater fineness of the microstructure and higher mechanical properties than were determined using the modifier that had the same chemical composition and was produced in the same way, but had the form of a rod.

Based on the results of the study, it was found that the form of the modifier affects the modification effect of the hypoeutectic silumin. The justification may be the nature of the modifier as a powder or a rod. By introducing a modifier in the form of a rod into the modified alloy, the process can be compared to the process of introducing an alloying element in order to change the chemical composition. Of course, as a result of introducing a very small amount of the modifier, a significant change in the chemical composition cannot occur. By introducing the modifier in the form of powder, its impact becomes more effective, most probably due to the greater number of places at which it affects the treated alloy. It should also be noted that the effect on the modification effect may depend on the size of the powder fraction. A fine powder can have a rod-like effect due to its rapid dissolution, while a powder with coarser grains can produce effective changes in micro-areas.

## 4. Conclusions

Based on the results of the research, it was shown that the form of the modifier is important as it determines the effectiveness of the modification process expressed by the microstructure and mechanical properties. After modification of AlTi1.5 in the form of powder, an increase in strength of 9 MPa, elongation of 0.7%, and hardness of 2 HB was obtained compared to the results of modification of the alloy with the modifier of the same composition that was introduced in the form of a rod. For AlB1.5, the following values were obtained: 11 MPa, 1.3%, and 3 HB. For AlSr, the following differences related to the modifier form were obtained: 3 MPa, 0.3%, and 0 HB. The greatest differences were obtained for the modifier in the form of powder cooled at 200 °C/s, which were as follows: 9 MPa, 1.0%, and 4 HB.

The most advantageous form of the modifier among the tested modifiers produced in the form of powder and rod was found to be the powder. For powder, a finer eutectic microstructure and resulting higher mechanical properties were obtained.

The tests showed that the granulation of the powder may also affect the microstructure of the alloy and the resulting mechanical properties. Determination of the optimal fraction of the modifier may be the subject of our next work.

## Figures and Tables

**Table 1:** Chemical compositions of tested hypoeutectic silumin AlSi7Mg.

Chemical Element	Si wt. %	Mg wt. %	Mn wt. %	Fe wt. %	Cu wt. %	Ni wt. %	Ti wt. %	B wt. %	Al wt. %
Average contents	7.24	0.30	0.26	0.13	0.10	0.006	0.00	0.00	bal.

## Data Availability

Not applicable.
